# An assessment of adherence to the WHO-delineated good manufacturing practice by the pharmaceutical companies in Kabul, Afghanistan

**DOI:** 10.1186/s12962-022-00348-1

**Published:** 2022-04-02

**Authors:** Rohullah Roien, Rajeev Shrestha, Kashikant Yadav, Akihiko Ozaki, M. Bashir Ahmadi, Yudai Kaneda, Yasuhiro Kotera, Binaya Sapkota, Sunil Shrestha

**Affiliations:** 1 Medical Research Centre, Kateb University, Kabul, Afghanistan; 2 Department of Pharmacy, District Hospital Lamjung, Besisahar, Province Gandaki Nepal; 3 Department of Research and Development, Samar Pharma Company Pvt. Ltd., Birgunj, Province Two Nepal; 4 Medical Governance Research Institute, Tokyo, Japan; 5 Afghanistan Pharmacists Association, Kabul, Afghanistan; 6 Hokkaido University School of Medicine, Sapporo, Japan; 7 Faculty of Medicine and Health Sciences, University of Nottingham, Nottingham, UK; 8 Department of Pharmaceutical Sciences, Nobel College, Affiliated to Pokhara University, Kathmandu, Province Bagmati Nepal; 9 Department of Pharmaceutical and Health Service Research, Nepal Health Research and Innovation Foundation, Lalitpur, Province Bagmati Nepal; 10 Nobel College of Health Sciences, Affiliated to Pokhara University, Kathmandu, Province Bagmati Nepal

**Keywords:** Afghanistan, Good manufacturing practice, Low-income countries, Pharmaceutical Industry

## Abstract

**Background:**

Afghanistan, a low-income landlocked country, is continuously suffering from domestic war and conflicts; the country struggles to provide quality healthcare services, including affordable medicinal products in the required quantity. Moreover, the quality standards of domestic pharmaceutical companies have not been established yet. One of the internationally recognized guidelines for monitoring manufacturing processes in pharmaceutical companies is Good Manufacturing Practice (GMP), recommended by World Health Organization (WHO). Therefore, this study aimed to assess whether a pharmaceutical company in Kabul, Afghanistan adheres to the GMP standards established by WHO.

**Method:**

A descriptive cross-sectional study was conducted to assess the WHO-delineated GMP compliance of 25 pharmaceutical companies in Kabul, Afghanistan. The inspection checklist was developed by Afghanistan's National Medicine and Healthcare Products Regulatory Authority (NMHRA) using the WHO-delineated GMP guidelines. In addition, direct observation, interviews with respective delegates, and documentation reviews were conducted to collect research data.

**Result:**

Only 38.33% (1.14 ± 1.08) of GMP contents were complied. Personnel 66.67% (2 ± 1.15) and materials 58.67% (1.76 ± 1.11) were the most commonly complied components, whereas the product recall 12.98% (0.39 ± 0.85), quality assurance 16.44% (0.49 ± 0.81) and quality control laboratory 28.35% (0.85 ± 1.12) were the least complied ones.

**Conclusion:**

None of the GMP components was fully adhered to by the pharmaceutical companies in Kabul, Afghanistan. Quality control and assurance should be implemented immediately, including validation and qualification practices.

**Supplementary Information:**

The online version contains supplementary material available at 10.1186/s12962-022-00348-1.

## Introduction

Lack of access to quality medicines (medicines that are free of harmful contaminants and adhere to required standards) is a global health challenge [1–3]. According to the World Health Organization (WHO), more than 10% of medicines available in the markets of low-income countries (LICs) are confirmed to be of poor quality [1]. Similarly, Ozawa et al. reported that 13.7% of medicines available in Asia are of poor quality [3]. Furthermore, about one-third of the WHO countries have no or less stringent drug regulatory systems [2]. Poor quality or substandard medicines can hamper the proper treatment of diseases, increase antimicrobial resistance, socioeconomic burden, cause doubts about medicines among clinicians and practitioners, and result in excessive overall pharmaceutical waste [1]. This is especially troublesome in LICs such as Afghanistan [1, 4].

The Islamic Republic of Afghanistan is a South Asian low-income landlocked county, which has experienced decades of political turmoil such as civil war and instability, severely affecting access to quality healthcare services, including pharmaceutical products [4]. Currently, the domestic pharmaceutical manufacturers are fulfilling only 5% of pharmaceutical demands within the country [5], and the rest of 95% is being imported from neighboring countries such as Pakistan, China, India, Iran, United Arab Emirates (UAE), and Turkey [6]. Similarly, according to the Afghanistan Pharmaceutical Profile 2011 report, the country lacks adequate access to essential qualitative medicines [7]. In addition, counterfeit, modified, and low-quality products have been found in the domestic market [4, 8]. It is estimated that about one billion dollars of pharmaceuticals are smuggled into Afghanistan annually [9]. Thus, Afghanistan's pharmaceutical sector is considered one of the least developed globally [4].

WHO has developed the minimal standards called Good Manufacturing Guideline (GMP) to ensure the consistent production of quality products starting from raw materials to equipment, premises, method audits and validations, human resource training, and the maintenance of sanitation and hygiene throughout the manufacturing process [10]. In addition, this guideline guarantees to reduce the potential risk of impurities and mishandling that cannot be removed or reversed in the testing and evaluation of the final product [10]. The guideline is now one of the most widely followed guidelines by pharmaceutical manufacturers worldwide [11].

The Afghan government also strives to comply with the WHO-delineated GMP standards to provide consumers with safe, quality, and affordable medicines. National Medicine and Healthcare Products Regulatory Authority (NMHRA), a national authority under the Ministry of Public Health (MoPH), has incorporated the GMP guidelines developed by WHO in 2017 into its five-year plan and set minimum mandatory standards (also applicable to imported products) for pharmaceutical companies to ensure compliance with GMP guidelines for quality manufacturing of pharmaceuticals and to prevent the marketing of counterfeit and substandard products [12, 13]. Previously, a pharmaceutical assessment study conducted in 2011 determines several checkpoints for strict compliance with GMP guidelines, including space, GMP-compliant equipment, technical documentation, skilled human resources and favorable policies [14].

Nevertheless, the exact scenario of GMP compliance of pharmaceutical companies in Afghanistan is not well explored in this regard, and there is a dearth of published information on the assessment of companies’ adherence to GMP practices. Accordingly, the current study aimed to assess the Afghan pharmaceutical companies’ compliance status to specific components of the GMP guidelines and recommend further improvements.

## Methodology

### Study design, setting, and period

A descriptive cross-sectional study was conducted from June to December 2018 to assess the GMP adherence scenario of pharmaceutical industries in Kabul, the capital city of Afghanistan. This study was conducted before the Taliban gained control of Afghanistan.

### Ethics approval

The research proposal was approved by the ethics committee of the Medical Research Centre at Kateb University (AF.KU. HREC.046 on May 05, 2018). The consent of the respective companies was obtained before conducting the research, and the research results were disseminated to the participating companies.

### Sampling method

In conducting this study, NMHRA provided the investigators with a list of companies in Afghanistan regulated by their organization. According to this list, the NMHRA regulated 100 companies in Afghanistan as of 2018. Among them, 30 companies manufactured cosmetic and hygienic products, while 70 companies produced pharmaceuticals. Of these 70 companies, 25 were included in the survey. Twenty-five companies were all located in Kabul.

### Study instrument

The questionnaire (Additional file 1: Appendix S1) consisted of two parts: a questionnaire on pharmaceutical products (such as industries’ names, number and type of manufactured items) and a questionnaire on the GMP inspection checklist defined by WHO. The GMP-checklist was developed by the technical team of NMHRA [10]. The checklist contained 66 items divided into 12 subdomains. The scoring to each item was done based on a Likert scale, where ‘1’ referred to non-compliance, ‘2’ to partial compliance, and ‘3’ to full compliance.

### Data collection and analysis

Two pharmacists with inspection and monitoring experience, were assigned to collect data from the field. Orientation on the checklist was given, followed by training on inspecting the industry's GMP compliance status and reporting the inspection results. The data collectors visited the pharmaceutical companies and collected the necessary information according to the checklist. In order to confirm the credibility of the information collected, interviews with pharmaceutical company representatives and reading of relevant documents were conducted to directly evaluate the performance and activities of the pharmaceutical companies. Additionally, after each pharmaceutical industry’s response, another field data collector cross-checked and reviewed completeness and accuracy of the collected data. Again, before entering the data into Microsoft (MS) Excel, the lead assessor reviewed and verified the data by communicating with industries’ representatives and reviewing the documents provided by the respective industries to assure the accuracy of data collected.

Data were entered into MS Excel 2010 and analyzed using descriptive statistics such as frequency and percentage. The values of individual indicators and overall domain indicators were presented to depict the conformance of specific manufacturers to the standard checklist and to explore areas that need improvement:

1. Scores of individual pharmaceutical industries on each domain were obtained.
2. From all 25 pharmaceutical companies, the average score and ratio were calculated by dividing the sum of the scores by the total number for each subdomain and domain of the GMP checklist.
3. A correlation test was conducted with Statistical Package for the Social Sciences (SPSS) version 25 to assess a relationship between the average score obtained by the pharmaceutical companies and the number of pharmaceutical items each company produced.

## Results

Among the 25 pharmaceutical companies, only six companies could manufacture more than 30 products, and only seven companies obtained more than 50% average score on GMP compliance. Altogether 9–70 types of different pharmaceutical items or medicinal products were manufactured in Kabul by the domestic industries. These products were available in a total of eight different dosage forms: solid (capsules, tablets, sprays), semi-solid (ointments), and liquid (syrups, oral, drops, topical) (Table 1).

**Table 1:** Summary of pharmaceutical industries in Kabul, Afghanistan

Pharmaceutical Companies	Types (number) of dosage form produced	Number of medicinal products manufactured	Average score obtained (%)
1	Capsule (1)	9	1.21 (40.33)
2	Oral solution, Syrup (2)	9	1.29 (43)
3	Syrup, Oral solution (2)	10	0.48 (16)
4	Capsule, Powder, Oral solution (3)	10	1.42 (47.33)
5	Oral solution (1)	11	0.58 (19.33)
6	Syrup, Tablet (2)	12	0.45 (15)
7	Drop, Oral solution (2)	15	0.27 (9)
8	Ointment, Drop, Syrup (3)	15	1.03 (34.33)
9	Tablet, Syrup (2)	17	0.64 (21.33)
10	Syrup, Oral solution (2)	17	2.45 (81.67)
11	Syrup (1)	20	0.7 (23.33)
12	Ointment (1)	23	2.26 (75.33)
13	Syrup, Ointment (2)	23	2.03 (67.67)
14	Syrup, Oral solution (2)	23	0.65 (21.67)
15	Ointment, Syrup, Oral solution (3)	24	0.52 (17.33)
16	Tablet, Syrup (2)	24	0.88 (29.33)
17	Tablet, Capsule, Syrup (3)	27	1.83 (61)
18	Tablet, Syrup (2)	27	0.95 (31.67)
19	Syrup, Powder (2)	28	0.61 (20.33)
20	Syrup, Ointment, Topical solutions (e.g., povidone-iodine and gentian violent) (3)	30	0.76 (25.33)
21	Tablet, Capsule (2)	30	1.03 (34.33)
22	Syrup, Powder (2)	30	1.98 (66)
23	Capsule, Syrup (2)	33	1.08 (36)
24	Syrup, Powder (2)	50	1.56 (52)
25	Syrup, Capsule, Tablet, Oral solution (4)	70	1.97 (65.67)
Total	8 types	587	1.15 (38.33)

Pearson's correlation between the average score and the number of pharmaceutical manufacturing companies did not show a significant association (p = 0.091).

Table 2 summarizes scores obtained by the 25 pharmaceutical industries of Kabul, Afghanistan, on the WHO-delineated GMP compliance checklist. The overall adherence to GMP domains by pharmaceutical companies was very poor. Only 38.33% (1.15 + 1.08) of domains were found to have adhered. Moreover, the companies did not meet the majority of the GMP domains. Among 12 domains of the WHO-delineated GMP checklist, only four domains obtained a greater score than 50%. The domain that best adhered to the guidelines was personnel (66.67%), followed by materials (58.67%), personal hygiene and sanitation (52.00%) and premises (50.67%). The least complied domains were product recall (12.98%), followed by quality assurance (16.44%) and quality control laboratory (QC lab) (28.35%) (Table 2). The average scores by pharmaceutical companies obtained for each domain and subdomain are shown in Additional file 2: Appendix S2.

**Table 2:** Summary of WHO-delineated GMP compliance of pharmaceutical industries

S.N	Main elements of GMP	Mean ± SD	Average score in percentage
1	Quality control laboratories	0.85 ± 1.12	28.35
2	Premises	1.52 ± 1.22	50.67
3	Personnel	2 ± 1.15	66.67
4	Documentation	1.41 ± 1.06	46.90
5	Product recall	0.39 ± 0.85	12.98
6	Training	0.94 ± 1.09	31.33
7	Personal hygiene & sanitation	1.56 ± 1.06	52.00
8	Equipment	1.35 ± 0.99	44.89
9	Materials	1.76 ± 1.11	58.67
10	Requirements for production	1.13 ± 1.18	37.50
11	HVAC and water system	1.22 ± 1.14	40.67
12	Quality Assurance	0.49 ± 0.81	16.44
Total		1.15 ± 1.08	38.33

*SD* Standard deviation, *HVAC* heating, ventilation and air-conditioning

**Fig. 1:**
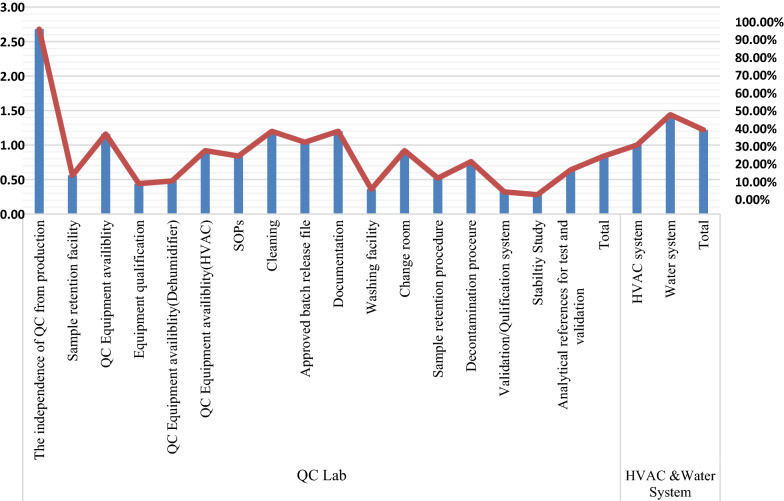
Scoring of each item on QC Lab, and HVAC and Water system domains of the GMP checklist

Figure 1 provides the detailed description of scores obtained on specific items under QC lab, Heating, Ventilation and Air-Conditioning (HVAC), and water system domains of the GMP checklist. Most industries had separate QC lab and production areas. However, stability testing, validation/qualification systems, cleaning facilities, and QC equipment (such as dehumidifiers) were relatively poorly installed. On the other hand, water facilities were much better than the HVAC system.

**Fig. 2:**
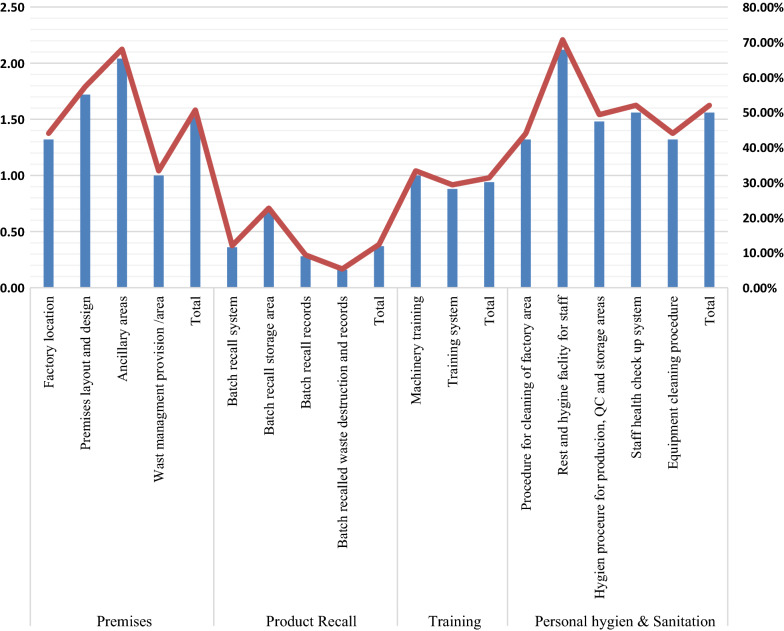
Scoring of each item on Premises, Product recall, Training, and Personal hygiene and sanitation domains of the GMP checklist

Figure 2 describes the detailed scores obtained on specific items under premises, product recall, training, and the GMP checklist's hygiene and sanitation domains. Among four specific premises components, the ancillary area was the most common in pharmaceutical industries, while the waste management provision/area was unavailable in most industries. Similarly, the batch recall storage area facility was comparatively better among other domains, while the batch recall waste destruction and records were worse among the four domains of the product recall component. Regarding the personal hygiene and sanitation component, rest and hygiene facility for staff was most commonly found to be available in most industries, but both cleaning procedure and equipment were found to have least complied with the GMP requirements.

**Fig. 3:**
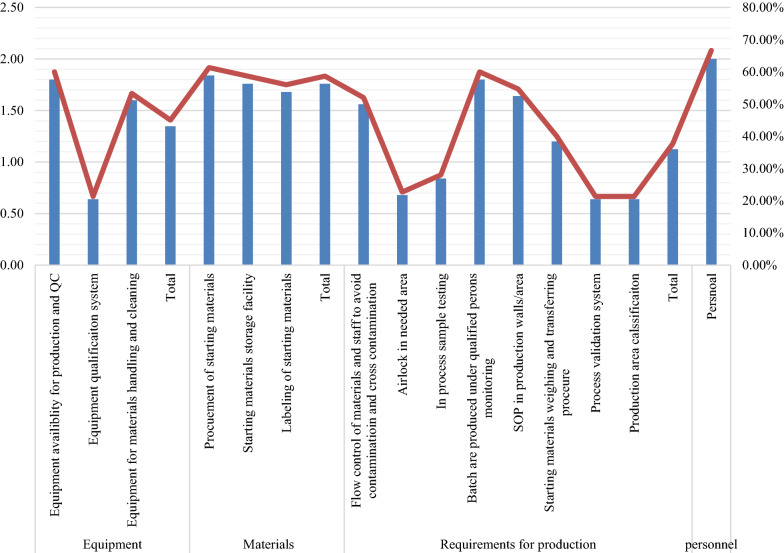
Scoring of each item on Equipment, Materials, Requirements for production, and personal domains of the GMP checklist

Figure 3 shows a graphical representation of the scores obtained in the equipment, materials, production requirements, and personnel domains of the checklist. Equipment for production and QC was relatively present, but the qualification system for equipment was relatively poor in the industries chosen for the study. Procurement, storage, and labeling of starting material scored similarly, with a slightly lower practice score for labeling. Similarly, the process validation system, production area classification, and airlock system showed poor adherence to the production requirements domain checklist.

In contrast, in most industries (n = 15), qualified personnel monitored the production process. The storage of Standard Operating Procedures (SOPs) at the production site was strictly followed after qualified personnel monitored the production process.

**Fig. 4:**
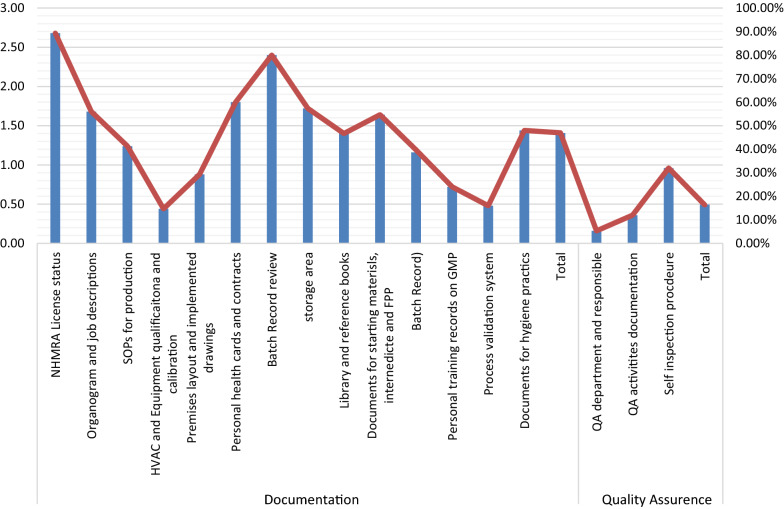
Scoring of each item on Documentation and quality assurance domains of the GMP checklist

Figure 4 shows the detailed scoring results for the documentation of the GMP checklist and the quality assurance (QA) domain. NMHRA's licensing and batch record review processes were followed more strictly than others. On the other hand, the documentation of the process validation system and the qualification and calibration of HVAC and equipment was very poor. For QA assurance, the self-inspection procedures were found to be more compliant than others.

## Discussion

The WHO-delineated GMP guideline is an internationally recognized and followed standard to justify and assure pharmaceutical industry quality procedures and credibility in manufacturing quality products. Pharmaceutical companies should follow the strict manufacturing standards outlined in the GMP standards established by WHO, as their main responsibility is to ensure and maintain the quality of pharmaceutical products for the period of their shelf life, even after they are sold and in the hands of consumers [14]. Based on an extensive search of documents in PubMed/Medline, ResearchGate, Google Scholar, Science Direct and others, to the extent of our knowledge, this is the first comprehensive study to investigate and assess the compliance of Afghan pharmaceutical companies with various aspects of the GMP standards established by WHO.

This study found that the pharmaceutical industry in Afghanistan mainly manufactures oral suspensions and external formulations such as cream and ointments. Only a few companies were manufacturing tablets and capsules formulations. Additionally, our study revealed that, on average, less than half (38.33%) of the GMP standards were adhered to by the companies. The most observed were personnel and materials, and the least were quality systems-related components such as product recall, QA, and QC. This suggests a relatively large number of pharmacy professionals and that the necessary resources are available to run the pharmaceutical industry. However, their performance was still inferior in terms of quality products manufacturing, probably due to the insufficient knowledge and awareness of pharmacy personnel or the least priority of the company owners toward maintenance of quality manufacturing practices. Moreover, the lower scores on the training component confirmed the knowledge gap of the workforce and sought the immediate necessity of relevant training to equip them with manufacturing and QA requirements.

A detailed analysis of the QC lab showed that less than half of the non-equipment components of the QC lab, independent of the manufacturing department, were GMP compliant. Specifically, the compliance rate of validation and qualification system, facility for sample retention, proper washing facility, and stability study of pharmaceuticals were less than 20%. The fact that these core contents of the QC lab are not well observed indicates the uncertainty of testing quality. In addition, improperly equipped and poorly functioning QC labs worsened the quality of manufactured products. Furthermore, product recall practices were rarely conducted, but only when instructed by the regulatory authorities during adverse event reporting. In addition, because there was no system for stability and quality evaluation testing after marketing or recall of substandard products [8], even at the time of importing, it was impossible to guarantee the quality of products sold or to detect products with deteriorated quality [4, 15]. Additionally, there is only one national-level QC laboratory in Afghanistan, and the government has begun the construction of four regional laboratories, which are not yet completed. Hence, the lack of laboratories may prevent strict monitoring of the products marketed and increase the possibility of counterfeit products in the markets.

Similarly, on investigating the premises-related components, waste management facilities were comparatively inferior in the companies, whereas the personnel hygiene of the staff was comparatively better. Although comparatively higher priorities were set to hygiene and sanitation of the workforce than the waste management system, the hazardous effects of inappropriate handling of pharmaceutical wastes would eventually affect human lives and the environment [16]. Therefore, equal priorities should have been given to preventing pharmaceuticals' cross-contamination from maintaining high levels of hygiene and sanitation of the workforce.

In the same way, the overall availability of personnel, equipment, and materials was relatively high. However, the qualification/calibration of equipment, method validation in production areas, proper arrangement of available production facilities with specialized airlock systems were poorly maintained. These results reflect the incapability of the pharmacy workforce or negligence of GMP guidelines by the employers, yielding quality-compromised products in the markets. Furthermore, since there was still no pharmacy council and pharmacist registration system in Afghanistan, this finding further indicates the immediate need to develop and implement a qualification evaluation system for pharmacists working in pharmaceutical industries along with relevant GMP training. Likewise, companies’ owners should ensure their manufacturing practices to follow the WHO-delineated GMP standards via various incentive schemes of the governments such as tax subsidies on importing equipment and raw materials.

NMHRA permits and batch record review processes were followed more strictly than the rest of the components. However, similar to all other components, the proper qualification, calibration of the HVAC system, and proper documentation of QA activities from the independent QA department were lacking in most industries.

Since 2020, the government of Afghanistan has restricted the import of 15 medicines in which the country was self-reliant [17]. However, the current failure of the pharmaceutical companies to adhere to the GMP standards established by WHO to manufacture and supply high-quality essential medicines to their target users suggests that the government is not capable of replacing the current high rate of drug imports [6]. Furthermore, although the government adopted a policy promoting domestic manufacturing with reduced imports [14], this study revealed that domestic pharmaceutical companies are not well prepared and prioritized, probably due to the lack of political stability, qualified pharmacy workforce, coordination, and communication within the MoPH and with other concerned stakeholders regarding strategic directions in uplifting the domestic companies, in addition to the lack of competency and awareness of administrators and pharmaceutical investors to strengthen the share of the domestic companies in the country.

## Strengths and limitations of the study

This study was conducted to understand the current status and scenario of GMP standards compliance of pharmaceutical companies in Afghanistan and to provide a useful guide for future research. However, due to the lack of previous reports on the status of GMP compliance in Afghanistan, this study could not visualize the promotion and demotion of performance. In addition, we could not consider the situation in the pharmaceutical industry outside of Kabul in this study. Furthermore, because this was a cross-sectional study, it lacked a follow-up study or causal assessment of non-compliance with GMP standards. Therefore, future nationwide surveys and intervention studies will probably provide a better glimpse of the status of compliance and improvement of GMP standards in companies based on the results of this study.

## Conclusion

Pharmaceutical companies in Afghanistan are required to immediately comply with the GMP standards set by WHO to ensure the quality of their products. However, it was found that none of the GMP contents were fully complied with, and only 38.33% of its contents were observed by the pharmaceutical companies in Kabul, Afghanistan. Quality control and assurance, including validation and qualification practices, should be implemented immediately. The proper utilization of available material (including equipment) and human resources can facilitate the manufacturers and policymakers in attaining the GMP standards. The concerned regulatory authorities and the private investors need to critically review their policies and strategies to strengthen the domestic pharmaceutical sectors to meet the country's demands for quality pharmaceutical services.

## Supplementary Information

**Additional file 1: Appendix S1.** GMP checklist for domestic pharmaceutical companies.

**Additional file 2: Appendix S2.** Details of the WHO-delineated GMP compliance of pharmaceutical industries.

## Data Availability

The datasets used and/or analyzed during the current study are available from the corresponding author on reasonable request.
