# Supplementary material for: An assessment of adherence to the WHO-delineated good manufacturing practice by the pharmaceutical companies in Kabul, Afghanistan
Source: Cost Eff Resour Alloc. 2022 Apr 2;20:17. doi: 10.1186/s12962-022-00348-1 (PMC8977029; doi:10.1186/s12962-022-00348-1)
Supplement: Supplementary file 1 — Additional file 1: Appendix S1. GMP checklist for domestic pharmaceutical companies. [file 12962_2022_348_MOESM1_ESM.docx]

**GMP Checklist for Domestic Pharmaceutical Companies**

Date:

Factory Name:

Production Line:

Finished Pharmaceuticals  Medical materials  Herbal Medicines  Cosmetics  Hygienic Products Laboratory Materials

Pharmaceutical Production Line Dosage Forms:

Total Number of Production Line:

| SN | Name of Assessor | Position | Sign |
| --- | --- | --- | --- |
|  |  |  |  |
|  |  |  |  |

Factory responsible confirmation:

| **S.N.** | **WHO GMP Element** | **Sub-elements** | | **Score** | **Remark** |
| --- | --- | --- | --- | --- | --- |
| 1 | QC Lab | 1 | Independence of QC department from the production | 0/3 |  |
|  |  | 2 | Sample retention facility | 0/3 |  |
|  |  | 3 | QC Equipment availability | 0/1/2/3 |  |
|  |  | 4 | Equipment qualification practice | 0/1/2/3 |  |
|  |  | 5 | QC Equipment availability (Dehumidifier) | 0/3 |  |
|  |  | 6 | QC Equipment availability (HVAC) | 0/3 |  |
|  |  | 7 | Availability of SOPs | 0/1/2/3 |  |
|  |  | 8 | Efficient cleaning service | 0/1/2/3 |  |
|  |  | 9 | Approved batch release file | 0/1/2/3 |  |
|  |  | 10 | Documentation practice | 0/1/2/3 |  |
|  |  | 11 | Washing facility | 0/3 |  |
|  |  | 12 | Change room facility | 0/3 |  |
|  |  | 13 | Appropriate sample retention procedure | 0/1/2/3 |  |
|  |  | 14 | Appropriate decontamination procedure | 0/1/2/3 |  |
|  |  | 15 | Validation/Qualification system | 0/1/2/3 |  |
|  |  | 16 | Stability Study facility | 0/1/2/3 |  |
|  |  | 17 | Analytical references for test and validation | 0/1/2/3 |  |
| 2 | Premises | 1 | Appropriate factory location | 0/3 |  |
|  |  | 2 | Appropriate premises layout and design | 0/1/2/3 |  |
|  |  | 3 | Ancillary areas facility | 0/1/2/3 |  |
|  |  | 4 | Waste management provision /area | 0/3 |  |
| 3 | personnel | 1 | Sufficient qualified personal | 0/1/2/3 |  |
| 4 | Documentation | 1 | NMHRA License status | 0/1/2/3 |  |
|  |  | 2 | Organogram and job descriptions | 0/1/2/3 |  |
|  |  | 3 | SOPs for production | 0/1/2/3 |  |
|  |  | 4 | Documentation of HVAC and Equipment qualification and calibration | 0/1/2/3 |  |
|  |  | 5 | Premises, layout and implemented drawings | 0/1/2/3 |  |
|  |  | 6 | Personal health cards and contracts | 0/1/2/3 |  |
|  |  | 7 | Batch Record review documentation | 0/1/2/3 |  |
|  |  | 8 | Batch Record review documentation | 0/1/2/3 |  |
|  |  | 9 | Proper recording of storage condition of storage area | 0/1/2/3 |  |
|  |  | 10 | Library and reference books | 0/1/2/3 |  |
|  |  | 11 | Documents for starting materials, intermediate and FPP | 0/1/2/3 |  |
|  |  | 12 | Documentation of Batch Record | 0/1/2/3 |  |
|  |  | 13 | Personal training records on GMP | 0/1/2/3 |  |
|  |  | 14 | Process validation system | 0/1/2/3 |  |
|  |  | 15 | Documents for hygiene practices | 0/1/2/3 |  |
| 5 | Product Recall | 1 | Batch recall system | 0/1/2/3 |  |
|  |  | 2 | Batch recall storage area | 0/3 |  |
|  |  | 3 | Batch recall records | 0/1/2/3 |  |
|  |  | 4 | Batch recall waste destruction and records | 0/3 |  |
| 6 | Training | 1 | Machinery training | 0/1/2/3 |  |
|  |  | 2 | Proper training system | 0/1/2/3 |  |
| 7 | Personal hygiene & Sanitation | 1 | Procedure for cleaning of factory and area | 0/1/2/3 |  |
|  |  | 2 | Rest and hygiene facility for staff | 0/1/2/3 |  |
|  |  | 3 | Hygiene procedure for production, QC and storage areas | 0/1/2/3 |  |
|  |  | 4 | Staff health checkup system | 0/1/2/3 |  |
|  |  | 5 | Equipment cleaning procedure | 0/1/2/3 |  |
| 8 | Equipment | 1 | Equipment availability for production and QC | 0/1/2/3 |  |
|  |  | 2 | Equipment qualification system | 0/1/2/3 |  |
|  |  | 3 | Equipment for materials handling and cleaning | 0/1/2/3 |  |
| 9 | Materials | 1 | Proper procedure for the procurement of starting materials | 0/1/2/3 |  |
|  |  | 2 | Starting materials storage facility | 0/1/2/3 |  |
|  |  | 3 | Labeling of starting materials | 0/1/2/3 |  |
| 10 | Requirements for production | 1 | Flow control of materials and staff to avoid contamination and cross-contamination | 0/1/2/3 |  |
|  |  | 2 | Airlock in the needed area | 0/1/2/3 |  |
|  |  | 3 | In process sample testing practice | 0/1/2/3 |  |
|  |  | 4 | Batch is produced under-qualified person monitoring | 0/3 |  |
|  |  | 5 | SOP in production walls/area | 0/1/2/3 |  |
|  |  | 6 | Starting materials weighing and transferring procedure | 0/1/2/3 |  |
|  |  | 7 | Process validation system | 0/1/2/3 |  |
|  |  | 8 | Production area classification | 0/1/2/3 |  |
| 11 | HVAC and water system | 1 | Availability of HVAC system | 0/1/2/3 |  |
|  |  | 2 | Proper water system | 0/1/2/3 |  |
| 12 | Quality Assurance | 1 | Provision of specialized QA department and responsibility | 0/3 |  |
|  |  | 2 | QA activities documentation | 0/1/2/3 |  |
|  |  | 3 | Self-inspection procedure | 0/1/2/3 |  |
