# Supplementary material for: An assessment of adherence to the WHO-delineated good manufacturing practice by the pharmaceutical companies in Kabul, Afghanistan
Source: Cost Eff Resour Alloc. 2022 Apr 2;20:17. doi: 10.1186/s12962-022-00348-1 (PMC8977029; doi:10.1186/s12962-022-00348-1)
Supplement: Supplementary file 2 — Additional file 2: Appendix S2. Details of the WHO-delineated GMP compliance of pharmaceutical industries. [file 12962_2022_348_MOESM2_ESM.docx]

**Appendix 2. Details of the WHO-delineated GMP compliance of pharmaceutical industries**

| **S.N.** | **Main Element of GMP** | **Sub-elements** | | **Average Score** | **Average Score in Percentage** |
| --- | --- | --- | --- | --- | --- |
| 1 | QC Lab | 1 | Independence of QC department from the production | 2.68 | 89.33 |
|  |  | 2 | Sample retention facility | 0.56 | 18.67 |
|  |  | 3 | QC Equipment availability | 1.21 | 40.28 |
|  |  | 4 | Equipment qualification practice | 0.50 | 16.67 |
|  |  | 5 | QC Equipment availability (Dehumidifier) | 0.48 | 16.00 |
|  |  | 6 | QC Equipment availability (HVAC) | 0.92 | 30.67 |
|  |  | 7 | Availability of SOPs | 0.84 | 28.00 |
|  |  | 8 | Efficient cleaning service | 1.20 | 40.00 |
|  |  | 9 | Approved batch release file | 1.04 | 34.67 |
|  |  | 10 | Documentation practice | 1.20 | 40.00 |
|  |  | 11 | Washing facility | 0.36 | 12.00 |
|  |  | 12 | Change room facility | 0.92 | 30.67 |
|  |  | 13 | Appropriate sample retention procedure | 0.52 | 17.33 |
|  |  | 14 | Appropriate decontamination procedure | 0.79 | 26.39 |
|  |  | 15 | Validation/Qualification system | 0.32 | 10.67 |
|  |  | 16 | Stability Study facility | 0.28 | 9.33 |
|  |  | 17 | Analytical references for test and validation | 0.64 | 21.33 |
|  |  | Subtotal | | 0.85 | 28.35 |
| 2 | Premises | 1 | Appropriate factory location | 1.32 | 44.00 |
|  |  | 2 | Appropriate premises layout and design | 1.72 | 57.33 |
|  |  | 3 | Ancillary areas facility | 2.04 | 68.00 |
|  |  | 4 | Waste management provision /area | 1.00 | 33.33 |
|  |  | Subtotal | | 1.52 | 50.67 |
| 3 | Personnel | 1 | Sufficient qualified personal | 2.00 | 66.67 |
| 4 | Documentation | 2 | NMHRA License status | 2.68 | 89.33 |
|  |  | 3 | Organogram and job descriptions | 1.68 | 56.00 |
|  |  | 4 | SOPs for production | 1.24 | 41.33 |
|  |  | 5 | Documentation of HVAC and Equipment qualification and calibration | 0.46 | 15.28 |
|  |  | 6 | Premises, layout and implemented drawings | 0.88 | 29.33 |
|  |  | 7 | Personal health cards and contracts | 1.80 | 60.00 |
|  |  | 8 | Batch Record review documentation | 2.40 | 80.00 |
|  |  | 9 | Proper recording of storage condition of storage area | 1.72 | 57.33 |
|  |  | 10 | Library and reference books | 1.40 | 46.67 |
|  |  | 11 | Documents for starting materials, intermediate and FPP | 1.64 | 54.67 |
|  |  | 12 | Documentation of Batch Record | 1.16 | 38.67 |
|  |  | 13 | Personal training records on GMP | 0.72 | 24.00 |
|  |  | 14 | Process validation system | 0.48 | 16.00 |
|  |  | 15 | Documents for hygiene practices | 1.44 | 48.00 |
|  |  | Subtotal | | 1.41 | 46.90 |
| 5 | Product Recall | 1 | Batch recall system | 0.41 | 13.64 |
|  |  | 2 | Batch recall storage area | 0.71 | 23.61 |
|  |  | 3 | Batch recall records | 0.28 | 9.33 |
|  |  | 4 | Batch recall waste destruction and records | 0.16 | 5.33 |
|  |  | Subtotal | | 0.39 | 12.98 |
| 6 | Training | 1 | Machinery training | 1.00 | 33.33 |
|  |  | 2 | Proper training system | 0.88 | 29.33 |
|  |  | Subtotal | | 0.94 | 31.33 |
| 7 | Personal hygiene and Sanitation | 1 | Procedure for cleaning of factory and area | 1.32 | 44.00 |
|  |  | 2 | Rest and hygiene facility for staff | 2.12 | 70.67 |
|  |  | 3 | Hygiene procedure for production, QC and storage areas | 1.48 | 49.33 |
|  |  | 4 | Staff health checkup system | 1.56 | 52.00 |
|  |  | 5 | Equipment cleaning procedure | 1.32 | 44.00 |
|  |  | Subtotal | | 1.56 | 52.00 |
| 8 | Equipment | 1 | Equipment availability for production and QC | 1.80 | 60.00 |
|  |  | 2 | Equipment qualification system | 0.64 | 21.33 |
|  |  | 3 | Equipment for materials handling and cleaning | 1.60 | 53.33 |
|  |  | Subtotal | | 1.35 | 44.89 |
| 9 | Materials | 1 | Proper procedure for the procurement of starting materials | 1.84 | 61.33 |
|  |  | 2 | Starting materials storage facility | 1.76 | 58.67 |
|  |  | 3 | Labeling of starting materials | 1.68 | 56.00 |
|  |  | Subtotal | | 1.76 | 58.67 |
| 10 | Requirements for production | 1 | Flow control of materials and staff to avoid contamination and cross-contamination | 1.56 | 52.00 |
|  |  | 2 | Airlock in the needed area | 0.68 | 22.67 |
|  |  | 3 | In process sample testing practice | 0.84 | 28.00 |
|  |  | 4 | Batch is produced under-qualified person monitoring | 1.80 | 60.00 |
|  |  | 5 | SOP in production walls/area | 1.64 | 54.67 |
|  |  | 6 | Starting materials weighing and transferring procedure | 1.20 | 40.00 |
|  |  | 7 | Process validation system | 0.64 | 21.33 |
|  |  | 8 | Production area classification | 0.64 | 21.33 |
|  |  | Subtotal | | 1.13 | 37.50 |
| 11 | HVAC and water system | 1 | Availability of HVAC system | 1.00 | 33.33 |
|  |  | 2 | Proper water system | 1.44 | 48.00 |
|  |  | Subtotal | | 1.22 | 40.67 |
| 12 | Quality Assurance | 1 | Provision of specialized QA department and responsibility | 0.16 | 5.33 |
|  |  | 2 | QA activities documentation | 0.36 | 12.00 |
|  |  | 3 | Self-inspection procedure | 0.96 | 32.00 |
|  |  | Subtotal | | 0.49 | 16.44 |
| 13 | **Total** | | | **1.15** | **38.33** |
